# Potential use of angiotensin receptor blockers in skin pathologies

**DOI:** 10.22038/IJBMS.2023.66563.14606

**Published:** 2023

**Authors:** Keshvad Hedayatyanfard, Azadeh Khalili, Hosein Karim, Soren Nooraei, Ehsan Khosravi, Nazgol-Sadat Haddadi, Ahmad-Reza Dehpour, Gholamreza Bayat

**Affiliations:** 1 Department of Physiology, Pharmacology and Medical Physic, School of Medicine, Alborz University of Medical Sciences, Karaj, Iran; 2 Cardiovascular Research Center, Alborz University of Medical Sciences, Karaj, Iran; 3 Department of Cardiology, School of Medicine, Alborz University of Medical Sciences, Karaj, Iran; 4 Student of School of Veterinary Medicine, Faculty of Veterinary Medicine, University of Shahrekord, Shahrekord, Iran; 5 Department of Veterinary Surgery, Science and Research Branch, Islamic Azad University, Tehran, Iran; 6 Department of Dermatology, University of Massachusetts School of Medicine, Worchester, MA, USA; 7 Department of Pharmacology, School of Medicine, Tehran University of Medical Sciences, Tehran, Iran

**Keywords:** Angiotensin II, Cancer, Keloid, Scar, Skin disease, Wound healing

## Abstract

Renin-angiotensin system (RAS) components such as angiotensin II, angiotensin receptors (AT_1_R and AT_2_R), and angiotensin-converting enzyme (ACE) are expressed in different cell types of the skin. Through AT1R, angiotensin II increases proinflammatory cytokines contributing to fibrosis, angiogenesis, proliferation, and migration of immune cells to the skin. In contrast, AT_2_R suppresses the effects mentioned above. Many studies show that angiotensin receptor blockers (ARBs) and angiotensin-converting enzymes (ACEi) reduce the proinflammatory cytokines and fibrogenic factors including transforming growth factor β (TGF-β), Connective tissue growth factor (CTGF), and IL-6. This review article provides a detailed research study on the implications of ARBs in wound healing, hypertrophic scar, and keloids. We further discuss the therapeutic potentials of ARBs in autoimmune and autoinflammatory skin diseases and cancer, given their anti-fibrotic and anti-inflammatory effects.

## Introduction

The intact epidermal layer has a vital role in maintaining the intradermal water amount and preventing the penetration of microorganisms or toxic agents. The formation of such a functional structure is regulated by a distinct balance between proliferation and differentiation processes (1). Renin-angiotensin system (RAS) components are located in cutaneous and subcutaneous layers, while RAS components are vital to skin pathophysiology, including inflammation, scar formation, fibrosis, and some skin malignancies (2). Notably, RAS is excessively activated during inflammation, has potent proinflammatory effects, increases vascular permeability, leads to edema, and involves cell proliferation, fibrosis, and inflammation (3). Active biological fragments of angiotensinogen, the mother peptide of RAS, including angiotensin II (Ang II), angiotensin 1-9 (Ang 1-9), angiotensin 1-7 (Ang 1-7), angiotensin III (Ang III), and angiotensin IV (Ang IV), as well as the classic angiotensin-converting enzyme (ACE) and the newer type (ACE2), have been considered new regulatory target molecules in normal skin physiology (2). Ang II receptors of AT_1_R and AT_2_R, which mediate opposing effects, are expressed in the keratinocytes, melanocytes, fibroblasts, and the deeper parts of the skin, including hair follicles and macrovesicle endothelial cells of sebaceous glands (2, 4). The pro-fibrotic, proinflammatory, and pro-proliferative effects of Ang II are predominantly mediated through AT_1_ receptors. Angiotensin receptor blockers are known anti-hypertensive drugs, while the growing body of evidence represents their functions beyond the vasculature system. 

In this review, we describe the application of ARBs in wound healing and skin fibrosis, focusing on the tendency to develop a topical route of delivery. We further elaborate on the potential therapeutic effect of ARBs in skin diseases, including psoriasis, cutaneous lupus erythematosus, and skin neoplasms. 

***Angiotensin Receptor Blockers (ARBs) and Wound Healing***

Wound healing is dynamic and occurs in three different phases: inflammation, proliferation, and remodeling. The inflammatory phase occurs 2-3 days after injury. Various inflammatory cells such as neutrophils and macrophages infiltrate the wound site and produce multiple cytokines and chemokines during the inflammatory phase, including platelet-derived growth factor (PDGF), transforming growth factor β  (TGF-β), interleukin 1 (IL-1), and tumor necrosis factor-alpha (TNF-α). These inflammatory mediators stimulate fibroblast cells to produce fibrin and extracellular matrix. The proliferation phase happens and lasts for three weeks. During the early stages of proliferation, the number of infiltrated inflammatory cells dwindle while collagen, granulation tissue, angiogenesis, and epithelialization accrete. Over the remodeling phase, collagen production and degradation are balanced and modified into mature collagen (5). Prolonged inflammation and excessive proinflammatory cytokine and matrix metalloproteinases (MMP_S_) production may induce impaired wound healing (2, 6). 

According to the literature on healing process alteration, the expression of RAS-related receptors is stage-dependent. Skin wound healing initiates an inflammatory phase followed by proliferation and remodeling with scar formation (2). In the initial proliferation phase, the expression of AT_1_R is rapidly increased. The same trend but at a slower rate also happen for AT_2_R. The process then continues with a distinct decline in the expression of AT_1_R but a dramatic up-regulation of AT_2_R. To a lesser extent, AT_1_R has increased during the remodeling phase. Hence, AT_2_R dominancy is apparent in the later phase (2, 7). 

Several studies have helped us find out the significant role of each receptor in modulating wound healing. In a series of studies, the effectiveness of the topical angiotensin II and angiotensin (1-7) was investigated in four wound models: diabetic rat and mouse full-thickness excisional wounds, rat cutaneous random flap, and partial thickness thermal injury model in the guinea pig. They found that angiotensin II and angiotensin (1-7) improve wound repair and increase keratinocyte and epidermal stem cell proliferation and the rate of epithelialization (8). There is some evidence elucidating the impact of ARBs on wound healing. Takeda’s findings revealed that stimulation of AT_1_R signaling accelerates keratinocyte migration in full-thickness skin wounds in rats and *in vitro* in the keratinocyte culture dish. However, AT_1_R blockers and the activation of AT_2_R reduce the speed of keratinocyte migration (9). The increase in keratinocyte migration is probably due to the generation of TGF-β and epidermal growth factor receptor (EGFR) phosphorylation (10, 11). Kamber *et al*. showed that oral administration of losartan (angiotensin II receptor blocker) for 14 days speeded up the epidermal repair in a streptozotocin-induced diabetic wound in mice (12). Research studies showed that valsartan has the highest skin penetration compared to other ARBs drugs (13), and the topical application of 1% valsartan (angiotensin II receptor blocker) gel was associated with a marked acceleration of wound healing in diabetic mice and aging pigs. Researchers showed that the wound healing rate was higher with topical valsartan versus topical losartan with the same formulation. They found that valsartan gel acts through AT_2_R because the healing effect disappeared in the AT_2_R null mice. On the other hand, the topical application of 5% captopril gel delayed the healing rate (14). 

Other studies using the valsartan 1% ointment (15) and new drug delivery methods in nano form have shown that daily topical 1% valsartan has reduced the Tgf-β signaling pathway and increased the acceleration of chronic wound healing in animal models (16).

***ARBs and scar***

Hypertrophic scar and keloids happen due to an impaired wound healing process leading to the overproduction of extracellular matrix. Hypertrophic scar and keloids differ in histology, incidence, appearance, location, and treatment. The incidence rate of hypertrophic scar is higher than keloids and may be up to 60% after surgery and regress after three months (17). On the other hand, the mechanism of keloid formation is still unclear, with various factors involved in the pathophysiology. 

Connective tissue growth factor (CTGF) (18), (TGF-β) (19), histamine (20), proinflammatory cytokine (IL-6) (21), renin-angiotensin system (22), vascular endothelial growth factor (VEGF) (23), MMP_S_ (24, 25), and tissue inhibitors of matrix metalloproteinases (TIMP) are among the long list of mediators that involve in scar formation. The interaction between the stromal and inflammatory cells and secretion of proinflammatory cytokines play a vital role in scar formation. TGF-β is one of the major factors in scar formation, which activates the type II receptor and phosphorylates the R-Smad proteins. The TGF-β/Smad signaling induces differentiation and migration of fibroblasts with consequent collagen production (26). It has been shown that the number of inflammatory cells (mast cell and macrophage) and proinflammatory cytokines (IL-6, IL-1β, and TGF-β) are increased in scar and keloid tissues (27). Therefore, chronic inflammation and delayed wound healing may contribute to scar progression.

Aside from the therapeutic effect of ARBs on the early stages of wound healing, ARBs are also found to prevent scar formation. We have already shown that AT_1_R and angiotensin II concentrations are higher in keloid and hypertrophic scar tissue than in normal skin in humans (22). Akershoek *et al.* findings also confirm up-regulation of AT_1_Rs in human scars (28). Later works of our group unveiled the anti-scar properties of ARBs in a clinical trial of thirty participants, including 20 cases who received losartan and ten control cases who were treated with a placebo. We found that application of topical losartan ointment at 5% twice a day significantly improved the Vancouver scar scale (VSS) scores, including vascularity, pigmentation, pliability, and height scores after three months (29). Zehang *et al.*(30) reported the same therapeutic effects with losartan ointment on the scar. Rsearch showed that oral ARBs and ACEIs reduced the scar width in the post-surgical scar in patients (31). Animal studies also support the anti-scar effects of ARBs and ACEIs (32). Both valsartan and enalapril were effective in preventing pathological scar formation in an experimental rabbit ear wound model. Interestingly, valsartan seemed to be more effective in reducing the fibroblast cell count and epithelial thickness (33). 

Ang II via AT_1_R increases the expression of IL-6, an essential mediator of scar formation (21). However, AT_2_ receptor stimulation with an AT_2_ receptor agonist (C21) has been shown to reduce TNF-α-induced IL-6 expression in human skin fibroblasts (34). Furthermore, TGF-β and CTGF, the two most essential mediators of scar formation, are produced following AT_1_R activation (4). ACEIs (such as ramipril and captopril) have been found to decrease the expression levels of TGF-β in full-thickness skin wounds of mice (35). ARBs (such as losartan) reduce CTGF expression in the lung fibrosis model in mice (36). Losartan also has been shown to inhibit the migration and contractile activity of human dermal fibroblasts and reduce scar formation in rats (32). The ratio between MMPs and TIMPs is essential in tissue fibrosis and scar pathogenesis. MMPs function in the collagen and extracellular matrix degradation, while TIMPs inhibit MMPs activity and thus involve hypertrophic scar and keloid development (25). Ang II has been found to increase collagen content accompanied by an increase in TIMP-1 expression in mice skin fibroblasts. These increases were inhibited by valsartan while being augmented by AT_2_R inhibition, representing that stimulation of AT_1_R and AT_2_R differentially regulates collagen production in mouse skin fibroblasts (37). 

***ARBs beyond wound healing and scar***

*Role of RAS components in skin cancer*

Angiogenesis and proliferation are two critical driving forces leading to cancer progression. According to the pivotal role of Ang II as a growth-stimulating factor in promoting angiogenesis and, therefore, tumorigenesis, the chemopreventive role of ARBs has been proposed in several types of cancer, such as nasopharyngeal carcinoma and breast cancer (38-41). Here is the evidence pointing to the involvement of Ang II in the progression and promotion of skin cancers. AT_1_R is highly expressed in squamous cell carcinoma (SCC) while being poorly expressed in basal cell carcinoma (BCC) (42). A randomized clinical trial showed that in a population at high risk of skin cancer, ACEI or ARB users had a lower incidence of BCC and SCC (41). Another survey that evaluated the incidence of skin cancer in renal transplant recipients revealed that the incidence of BCC and SCC cancers dropped with the consumption of ACEI or ARB (43). 

Moreover, the inhibitory effect of losartan on the growth of murine melanoma confirms the oncogenic properties of AT_1_R (44). Hematogenous metastasis in mouse melanoma cells following administration of a non-selective receptor agonist of AT_1_R and AT_2_R was suppressed with valsartan (45). AT_1_R had an essential role in the angiogenesis and growth of melanoma tumor cells engrafted in mice. Although angiogenesis was prominent in wild-type (WT) mice, it was reduced in AT_1_R-deficient mice, and TCV-116, a selective AT_1_R blocker, decreased melanoma growth and angiogenesis in WT mice (46). Besides the evidence regarding the protective effects of ARBs, the oncogenic properties of AT_1_Rs are confirmed by the down-regulation of the AT_1_R encoding gene using miRNA miR-410, which consequently suppressed tumor growth and migration (47). Although AT_1_R is known as an oncogenic receptor with a proliferative effect (2, 7-9), a recent study showed the AGTR1 (encoding AT_1_R) suppressor effect following ectopic expression of AGTR1 (encoding AT_1_R) in melanoma cell lines lacking endogenous expression of AT_1_R (47). 

Some studies have shown the opposite effects of ARBs and ACEi drugs in the occurrence of skin cancer(48), which may be due to the different administration methods and dosage. 

***ARBs in autoinflammatory/autoimmune skin diseases***

Ang II via AT_1_R has proinflammatory effects mediated through several chemokines and cytokines and induces specific inflammatory signaling pathways (reactive oxygen species (ROS), nuclear factor-κB, IL-6, and TGF-β) (21, 49-51). Hence, it could contribute to several autoinflammatory and autoimmune diseases like systemic lupus erythematosus (SLE) (52). 

Psoriasis is an autoinflammatory skin disease associated with high serum levels of ACE (53, 54) and hyperactivity of components of RAS in the skin (55). Polymorphism of the *ACE I/D* (insertion (I) and deletion (D)) gene is reported in psoriatic patients which might contribute to the high levels of serum ACE (56). Overproduction of tissue ACE and Ang II levels negatively affects the balance between keratinocyte proliferation and differentiation (2). In our previous animal study of imiquimod-induced psoriatic inflammation in mice, we found that the topical administration of losartan 1% significantly decreased the psoriasis area and severity index (PASI) score accompanied by reduced levels of IL-17a, Ang II, and AT_1_R expression (57). Previous studies showed that Ang II increases the level of IL-17 and losartan decreases Th17 infiltration cells (58, 59). Although there are data on the negative effect of ARBs on psoriatic skin lesions (60-62), we still need to assess the impact of RAS-modifying therapies on psoriasis precisely.

Systemic lupus erythematosus is a chronic autoimmune disease involving the skin in many cases (63). ACE gene polymorphisms are associated with SLE pathogenesis (64). however, data supporting the therapeutic effects of these classes of drugs in cutaneous lupus is limited. Recently, Soto *et al*. have shown the therapeutic effect of losartan and lisinopril as well as angiotensin (1-7) [A(1-7)], Nor-Leu-3 angiotensin (1-7), the other RAS-modifying reagent, in the MRL*-lpr* mouse model of SLE. Their findings demonstrated that the RAS-modifying therapies significantly reduced the onset and severity of skin rashes (65). Overall, the limited presented data set on the effectiveness of ARBs on the cutaneous lesion of SLE is not sufficient to decide the relative conclusion in this regard, and it needs more experimental and clinical data.

Epidermolysis bullosa is a genetic skin disorder associated with chronic inflammation and scarring. There is a case report of the therapeutic effect of losartan on epidermolysis bullosa in a 6-year-old patient (66). It also has been shown that losartan ameliorates dystrophic epidermolysis bullosa in recessive dystrophic epidermolysis bullosa (RDEB) mice by reducing the TGF-β signaling and thus halting fibrosis. Interestingly, their findings of proteomics analysis reveal the decrease of multiple proteins related to tissue inflammation (67). Further studies are needed to evaluate the effect of losartan and other ARBs on epidermolysis bullosa. Other studies have shown losartan to have beneficial effects in the treatment of Myhre syndrome characterized by connective tissue disorders and skin thickening (68).

## Conclusion

This review study aimed to evaluate the role of RAS in dermatology and skin disorders. It has been clear that Ang II, throughout AT_1_R, increases angiogenesis, inflammation, migration of fibroblasts, keratinocytes, and melanocytes, and consequently enhances fibrosis and scar formation in the skin (4). Some research indicated that the expression of VEGF was reduced in knockout (AT_1_a^-/-^) mice, and AT_1_R antagonist decreased VEGF and the wound healing process (69). On the other hand, activation of AT_2_R alleviates angiogenesis, and the release of proinflammatory cytokines (IL-6, TNF-α, and TGF-β) contributes to skin fibrosis (34, 70). 

Based on previous studies, it seems that ARBs and ACEi drugs can inhibit the production of excessive inflammation in skin wounds and accelerate the healing of chronic wounds such as diabetic ulcers (12). Inflammation and pro-inflammatory cytokines play an important role in collagen production and scar formation, the use of ARBs and ACEi drugs may have a therapeutic role in reducing scars.

Furthermore, there is a growing body of evidence in favor of the therapeutic effects of ARBs in the context of autoimmunity and autoinflammation. ARBs are commonly used to inhibit AT_1_Rs; however, agonists and antagonists for AT_2_R have not yet been approved for clinical use. The compound 21 (C21) is an agonist of non-peptide AT_2_R owned by vicore pharma (Sweden) Which is in phase II clinical trials with approved anti-inflammatory effects.

Further studies recommended using AT_2_R agonists for dermatological diseases. The topical formulation of ARBs would be a better therapeutic option for skin disorders. Still, the epidermal layers prevent drugs from penetrating deeper into the skin (71). The solubility, molecular weight, and lipophilicity of the ARBs and their potency and affinity for AT_1_Rs are different (72). Therefore, it may be necessary to use novel drug delivery carriers or methods for increased penetration into deeper layers of the skin (73). 

## Authors’ Contributions

K H, GH B, H K, S N, E KH, A D, and A KH participated in all aspects of the main file draft. N S participated in the revision of the article.

## Conflicts of Interest

The authors declare that there are no conflicts of interest.
